# Cutaneous lesions in the setting of disseminated *Cryptococcus neoformans* infection

**DOI:** 10.4102/sajhivmed.v22i1.1315

**Published:** 2021-10-29

**Authors:** Mithra John, Stacey Norsworthy, Lauren Richards

**Affiliations:** 1 Department of Health, Internal Medicine, Helen Joseph Hospital, Johannesburg, South Africa

A 38-year-old HIV-positive South African man presented with a 3-week history of headache and worsening widespread cutaneous lesions. He had recently started antiretroviral treatment (ART), was virologically suppressed and had a CD4 T-lymphocyte count of 21 cells/µL.

**FIGURE 1:**
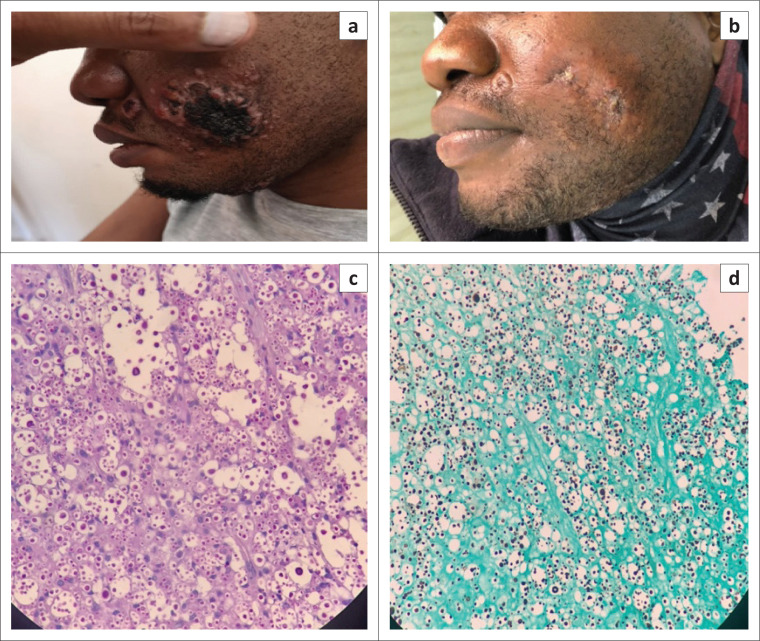
(a) A 4-cm, centrally necrotic plaque on the patient’s left cheek with a smaller papule adjacent to it; (b) significant improvement of the same lesions following 5 weeks of therapy; (c) periodic acid–Schiff and (d) Grocott–Gomori stained sections showing numerous fungal organisms with a large clear capsule, in keeping with *Cryptococcus neoformans* (original magnification, 40×).

Clinically, the patient had ulcerated, centrally necrotic plaques and papules found mainly on the face and scalp, with lesser involvement of the torso and limbs. The largest lesion was noted to be on his left cheek and measured 4 cm in diameter (Figure 1a). Additionally, the patient displayed signs of meningitis and a right cranial nerve VI palsy on neurological examination.

A diagnosis of disseminated *Cryptococcus neoformans* infection was made based on a positive serum and cerebrospinal fluid *Cryptococcus* antigen and a skin punch biopsy. Histological evaluation of the skin biopsy specimen revealed numerous fungal organisms with a large clear capsule and narrow-based budding on both periodic acid–Schiff and Grocott–Gomori’s methenamine silver staining in keeping with *C. neoformans* (Figure 1c and 1d). This was further confirmed on fungal culture of the biopsy specimen.

The patient received amphotericin B deoxycholate and flucytosine for 7 days, as well as daily therapeutic lumbar punctures to relieve raised intracranial pressure. He was then transitioned to oral fluconazole, and ART was reintroduced 5 weeks later. The patient responded well to treatment, with significant improvement to his skin lesions (Figure 1b).

